# Properties of skyrmions and multi-quanta vortices in chiral *p*-wave superconductors

**DOI:** 10.1038/srep17540

**Published:** 2015-12-03

**Authors:** Julien Garaud, Egor Babaev

**Affiliations:** 1 Department of Theoretical Physics and Center for Quantum Materials KTH-Royal Institute of Technology, Stockholm, SE-10691 Sweden

## Abstract

Chiral *p*-wave superconducting state supports a rich spectrum of topological excitations different from those in conventional superconducting states. Besides domain walls separating different chiral states, chiral *p*-wave state supports both singular and coreless vortices also interpreted as skyrmions. Here, we present a numerical study of the energetic properties of isolated singular and coreless vortex states as functions of anisotropy and magnetic field penetration length. In a given chiral state, single quantum vortices with opposite winding have different energies and thus only one kind is energetically favoured. We find that with the appropriate sign of the phase winding, two-quanta (coreless) vortices are always energetically preferred over two isolated single quanta (singular) vortices. We also report solutions carrying more flux quanta. However those are typically more energetically expensive/metastable as compared to those carrying two flux quanta.

Chiral *p*-wave superconducting state is an exotic state that, in addition to usual U(1) gauge symmetry, spontaneously breaks time-reversal symmetry. Higher broken symmetries there, implies a much richer spectrum of topological excitations as compared to conventional superconducting and superfluid states. Chiral *p*-wave pairing is realized in the A-phase of superfluid ^3^He, were variety of complex topological defects were investigated^1^,^2^,^3^,^4^,^5^,^6^,^7^,^8^. In a superconducting *p*-wave state, due to the coupling to the vector potential, topological defects exhibit different properties. This coupling affects their energy and determines their role in the magnetic properties of such superconductors. Layered perovskite superconductor Sr_2_RuO_4_ is a candidate material where various experimental evidences suggest possible realization of a *p*-wave superconducting state^9^,^10^. Similar models were also considered in connection to the superconducting state of heavy fermion compound UPt_3_ 11,12 (see e.g.^13^,^14^ for recent discussion of superconducting state in that material).

Spontaneous breaking of time-reversal symmetry for chiral *p*-wave state, implies the existence domain walls that separate regions with two different time-reversal symmetry broken (TRSB) ground-states. These domain walls, support spontaneous supercurrent that can generate magnetic fields^15^,^16^,^17^,^18^,^19^,^20^. The domain walls in chiral *p*-wave superconductors could be created via Kibble-Zurek mechanism, and these properties can be used for their control^21^. However, in Sr_2_RuO_4_ no indication of such a field was found in magnetic imaging microscopy experiments^22^,^23^,^24^. Besides domain walls, chiral *p*-wave superconductors feature rich spectrum of topological defects including various vortices and skyrmions. Note that Skyrmions were also discussed in other kinds of p-wave superconductors^25^,^26^. Related topological defects were also discussed in the context of heavy fermion superconductor UPt_3_ 27.

In zero field, both chiral (ground-)states are degenerate in energy and this degeneracy is lifted by an externally applied magnetic field along the *c*-axis. For a given sign of the magnetic field parallel to the *c*-axis, only one of the chiral states is stable and the time-reversed state is energetically penalized. Likewise, vorticity of the superconducting condensates lifts the degeneracy between both chiral states. When the dominant component forms a vortex, it induces the time-reversed (subdominant) chiral component, in the vicinity of the core. The winding of the induced component is not independent of that of the dominant component. It has a 4*π* winding of the relative phase between components, that follows from the Cooper pairs having nonzero internal orbital momentum^28^. Since the magnetic field lifts the degeneracy between chiralities, vortices and anti-vortices have different properties^29^.

It is experimentally seen that in an applied external field, Sr_2_RuO_4_ exhibits vortex lattices with square symmetry at high fields^30^,^31^,^32^, and a transition to triangular lattice in lower fields^32^,^33^. Earlier theoretical calculations based on Ginzburg-Landau model for chiral *p*-wave superconductivity in Sr_2_RuO_4_ 34,35,36, are consistent with these observed transitions of the vortex lattice structure.

Besides single-quanta vortices, there also exists vortices carrying multiple quanta of the magnetic flux and that, as they are coreless, are essentially different from single-quanta vortices. For example as discussed in more details below, the component induced by a doubly quantized vortex in the dominant component has zero winding in subdominant one^29^. In this paper we demonstrate that the two-quanta (coreless) vortices, which can also be denoted as skyrmions, are energetically favoured as compared to (isolated) single-quanta vortices. Earlier works in the context of UPt_3_, even claim that lattices of similar two-quanta vortices may be energetically favoured as compared to those of single quanta^37^,^38^. The possible existence of lattices of different coreless vortices carrying single flux quantum in UPt_3_ was also discussed recently^14^. It was also recently shown in the context of Sr_2_RuO_4_, based on solutions of microscopic Eilenberger equations, that lattices of two-quanta vortices are favoured for certain parameter sets^39^. Yet, such lattices of two-quanta vortices were never observed in Sr_2_RuO_4_. This motivates this work to further investigate the thermodynamic stability of skyrmions for broad parameter range.

In a previous work^40^, we reported isolated skyrmion solutions in a model for chiral *p*-wave superconductor. For the studied case of one of the chiralities, skyrmions can be energetically favoured as compared to vortices [Note that the Ginzburg-Landau model which was used in ref. 40 had slightly different coefficients in the potential terms compared to the standard GL model which follows from the weak-coupling mean-field theory. In this paper we use the same model as in ref. 36]. The skyrmions carrying two flux quanta are directly related to the two-quanta vortices mentioned above. However it was also demonstrated in ref. 40 that there are (meta-)stable skyrmions carrying larger number of flux quanta. In this model the energy and structure of vortices and skyrmions depends on the chirality. Equivalently, for a given chiral state, vortex/skyrmion solutions are not the same as anti-vortex/anti-skyrmion. It thus calls for further investigation of vortex and skyrmion solutions (carrying two and more quanta), which we present below.

## Model

In the coordinate system where the crystal anisotropy axis is 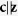, the order parameter of the *p*_*x*_ + *ip*_*y*_ state is described by a complex two-dimensional vector ***η*** = (*η*_*x*_, *η*_*y*_)^10^,^11^,^41^. Introducing the chiral order parameter components 
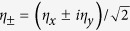
, the dimensionless Ginzburg-Landau free energy reads as (see e.g.^34^,^35^,^36^):


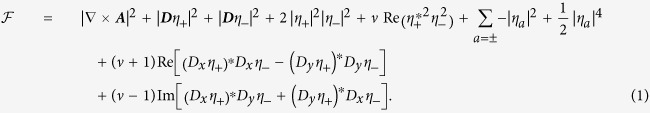


There we use dimensionless units were the free energy is normalized to the condensation energy, and the lengths are given in units of 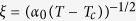. The magnetic field 
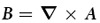
 is in units of 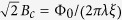. In these units, the gauge coupling *g* that appears in the covariant derivative ***D*** = **∇** + *ig**A*** is related to the Ginzburg-Landau parameter *g*^−1^ = *λ*/*ξ*. The free energy (1) was derived from the weak coupling microscopic theory^34^,^35^. The anisotropy parameter *v* determines the anisotropy in the *xy*-plane (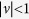 for the energy to be positively defined). It measures the tetragonal distortions of the Fermi surface, which has cylindrical geometry for *v* = 0, and is defined as 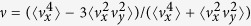 (where 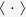 denote average over the Fermi surface). In the model Eq. (1), the dependence on the third coordinate is not considered (i.e. assuming two-dimensional system or translational invariance along *z*-axis).

The ground-state that minimizes the potential terms in (1) is degenerate and the solutions are (*η*_+_, *η*_−_) = (1, 0) and (0, 1). The theory (1) is invariant under the (discrete) time-reversal operations 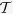, as 
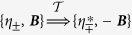
. This invariance is spontaneously broken by the ground-state. The spontaneous breakdown of the discrete time-reversal symmetry dictates that the theory allows domain wall solutions that interpolate between regions with different ground-states. Such domain walls, rather generically created during phase transition where the discrete symmetry is broken^21^, carry a magnetic field perpendicular to the *xy*-plane^17^,^18^. The discrete degeneracy of the ground state is lifted by the magnetic field. Thus, depending on the direction of the external field, only one state is stable. Likewise, the vorticity of the superconducting condensates lifts the degeneracy between chiral (ground-)states.

As the components *η*_+_ and *η*_−_ behave differently for different sign of the winding, a complete study requires to consider both situations of counter-clockwise (positive) and clockwise (negative) vorticities. Note that this is equivalent to considering only positive vorticity but for both chiral states. For example, the configuration with a winding *n*_+_ = +1 on the ground-state (*η*_+_, *η*_−_) = (1, 0) can be obtained by applying the time-reversal operation on the configuration whose ground state is (*η*_+_, *η*_−_) = (0, 1) with the winding *n*_−_ = −1. In the following, we choose to fix the dominant component of the order parameter to be *η*_−_ [i.e. the ground state is (*η*_+_, *η*_−_) = (0, 1)] and thus need to investigate both positive and negative vorticities.

The asymptotic vorticity of the dominant component *η*_−_ determines the sign of *B*_*z*_, as well as the vorticity of the subdominant component *η*_+_ 35, according to:


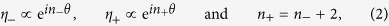


where *θ* is the polar angle. The relative phase between *η*_+_ and *η*_−_ (2), follows from the internal orbital momentum of Cooper pairs. In the Ginzburg-Landau model (1), this is the structure of mixed gradient that constraints the relative phase. Note that since the subdominant component *η*_+_ vanishes asymptotically [i.e. it recovers its ground state value *η*_+_ = 0], the winding *n*_+_ can be located only in a close vicinity of the vortex core. Note also that the winding of the subdominant component does not affect the overall flux quantization, because the density of that component vanishes away from the vortex. From (2) it is rather straightforward to see that the vortex with the vorticity (*n*_+_, *n*_−_) = (+3, +1) and the (anti-)vortex with (*n*_+_, *n*_−_) = (+1, −1) have different core structures and thus different energy.

## Results

**Figure 1:**
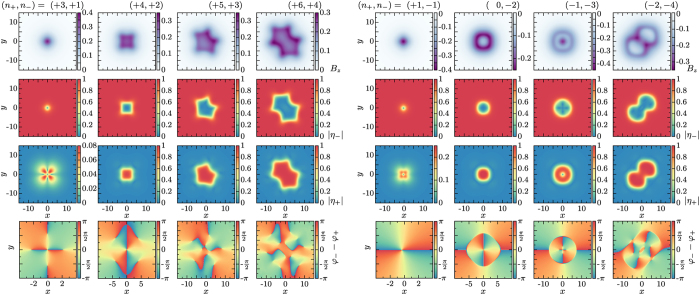
Vortex states for the windings (*n*_+_, *n*_−_) of the components *η*_+_ and *η*_−_. Note that the winding of the dominant component (here *η*_−_), specifies the flux carried by the vortex configuration. The parameters of the Ginzburg-Landau functional are *g* = 0.3 and *v* = 0.2. The first line shows the magnetic field ***B***, while second and third line respectively display 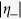 and 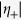. The fourth line shows the relative phase *φ*_−_ − *φ*_+_ between *η*_+_ and *η*_−_. Winding of the relative phase indicates the position of the cores of *η*_+_ and *η*_−_. The first block shows vortex solutions carrying one to four flux quanta with *B*_*z*_ > 0, while the second block shows the corresponding vortices with *B*_*z*_ < 0.

In order to investigate the energetic properties of vortex matter, the fields *η*_±_ and ***A*** are discretized using a finite-element framework^42^ and the free energy (1) is minimized using an nonlinear conjugate gradient algorithm (see the section Methods for details). In simulations of chiral *p*-wave superconductors on a finite domain, a special attention is required for boundary conditions in order to yield edge currents (see for example discussions in^19^,^41^,^43^). Here, we are interested in the intrinsic energetic properties of isolated defects. Thus vortex configuration is created by an initial guess and placed them at the center of a large domain, with open boundary conditions, letting the fields freely reach the ground-state. By choosing a sufficiently large domain, this ensures that within numerical accuracy vortices will not interact with boundaries and thus we are able to probe their intrinsic structure and energy properties, without effects of boundary conditions. As it specifies the topological sector, a starting configuration with a given winding *n*_−_ of the dominant component *η*_−_ leads, after convergence of the algorithm, to a configuration that behaves as expected from (2). We systematically construct vortex solutions carrying one to four flux quanta for parameter space defined by wide range of values of the *g* and *v*. Figure 1 shows typical vortex solutions with different vorticities. Along this paper we also refer to vortices carrying multiple flux quanta, as *skyrmions*. The reason for that terminology is that they have additional topological properties, as compared to single quanta vortices. This is explained in more details by the end of the paper.

The first and second blocks in Fig. 1 respectively show vortex solutions with *B*_*z*_ > 0 and *B*_*z*_ < 0. Vortices carrying from one to four flux quanta are displayed within each block. As expected from Eq. (2), single winding of the dominant component induces core structure of the subdominant component with different winding depending on that of *η*_−_ (see the first column of each block). It is instructive to consider the last row in Fig. 1, that displays the relative phase between *η*_−_ and *η*_+_. In agreement with (2), asymptotically the relative phase *φ*_−_ − *φ*_+_ = −2*θ*, reflecting the orbital angular momentum difference between *η*_−_ and *η*_+_. Moreover, the relative phase also indicates the position of the singularities of the components of the order parameter. Remarkably single quanta vortices are singular defects, since singularities in both components overlap (and thus *η*_+_ = *η*_−_ = 0). On the other hand, since both components never simultaneously vanish, two-quanta vortices are coreless defects. Interestingly the *n*_−_ = −2 configuration features a *π* jump of the relative phase when going outward from the vortex core. Inside the vortex core the time-reversed chiral state (*η*_+_, *η*_−_) = (1, 0) is induced, while the (*η*_+_, *η*_−_) = (0, 1) state is recovered asymptotically. The two quanta vortices thus feature a ringlike domain wall when going away from the center. The *π* jump of the relative phase for the *n*_−_ = −2 is located at this domain wall.

Like in conventional superconductors, the magnetic field for single quanta vortices is localized at the vortex core and screened at length scales determined by the penetration depth *λ*. Interestingly, the magnetic field for two-quanta vortices, and especially for *n*_−_ = −2, is not homogeneously distributed in the core. Rather it is localized at a given distance from the center and spread along the ring of the domain wall. Note that similar vortex configurations were also found to exist in the context of two-component model with 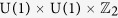 symmetry^44^. The ring-like distribution of the magnetic field for the two-quantum vortex can be understood as follows: ***B*** outside the vortex is screened by the (partial) currents in *η*_−_ that run counter-clockwise, while inside the vortex currents in *η*_+_ are responsible for the screening. Since *η*_+_ vanishes away from the vortex core, it cannot contribute to the screening asymptotically. Conversely, *η*_−_ vanishes at the vortex core and this is the induced subdominant component *η*_+_ that screens ***B*** close to the center of the vortex core. The reason it can contribute to screening (inside the vortex) without having vorticity on its own, is only due to supercurrent produced by the vector potential (like the Meissner currents on the boundary of ordinary superconductors). Since those currents circulate clockwise, they compensate with the currents in *η*_−_ so that at a certain distance (at the domain wall) there is no screening current. The magnetic field is thus localized at the domain wall. Although the core structure of single-quanta vortices are different depending on the sign of *n*_−_, their profile of the magnetic field looks quite similar. When considering vortices with 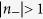, both the core structure and the magnetic field profile are strikingly different and the skyrmions with negative *n*_−_ do not resemble those with positive *n*_−_. Apart from the *n*_−_ = −2, −3 skyrmions, the configurations that carry multiple flux quanta are far from being axially symmetric. Note that the *n*_−_ = −4 skyrmions resembles as some kind of bound state of two *n*_−_ = −2 skyrmions. As we will discuss below, this makes their decay into two *n*_−_ = −2 vortices rather easy.

**Figure 2:**
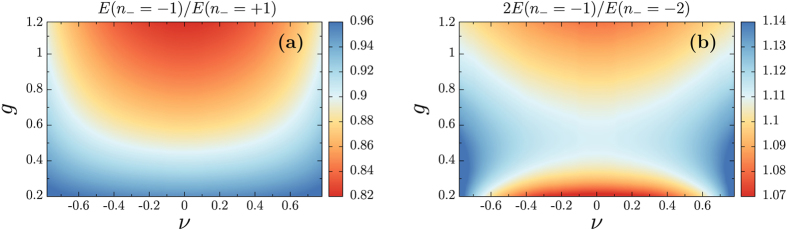
Left panel shows the ratio of the energies *E*(*n*_−_ = −1) of *n*_−_ = −1 single quanta vortices and *E*(*n*_−_ = +1) of *n*_−_ = +1 vortices, as functions of the anisotropy parameter *v* and of the gauge coupling *g*. This ratio is always smaller than 1 implying that (*n*_+_, *n*_−_) = (+1, −1) are always less energetic than (*n*_+_, *n*_−_) = (+3, +1). The right panel displays the ratio of the energies 2*E*(*n*_−_ = −1) of two *n*_−_ = −1 single-quanta vortices and *E*(*n*_−_ = −2) of one *n*_−_ = −2 vortex, as functions of the anisotropy parameter *v* and of the gauge coupling *g*. This ratio is always larger than 1 implying that two-quanta vortices are less energetic than two isolated single-quanta vortices.

Since the core structure is different, it is quite natural to expect that, unlike in conventional superconductors, vortices with opposite winding (and thus opposite directions of the magnetic field) are non-degenerate in energy. The (*n*_+_, *n*_−_) = (+3, +1) vortex has more total vorticity than the (*n*_+_, *n*_−_) = (+1, −1). Thus one could naively expect that the *n*_−_ = −1 vortices would be favoured as compared to *n*_−_ = +1. We systematically compared the energies of both single-quanta vortices for all values of the anisotropy parameter *v* and of the gauge coupling *g*. The diagram in Fig. 2(a), shows that the ratio of the energies of the single quanta vortices with *n*_−_ = −1 and *n*_−_ = +1, is always less than one. This implies that vortices *n*_−_ = −1 are always energetically favoured, as compared to those with *n*_−_ = +1. The first critical field of a vortex carrying a flux Φ is H_*c*1_ = *E*/2Φ, where *E* is its energy. As a result, Fig. 2(a) also implies that the *n*_−_ = −1 vortices also have lower first critical field H_*c*1_ in agreement with refs 36,45. Although both *n*_−_ = ±1 are perturbatively stable (i.e. they are minima of 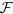), only *n*_−_ = −1 is absolutely stable.

Note that the naive estimates based on counting the total vorticity provide the correct picture that (*n*_+_, *n*_−_) = (+1, −1) vortices are less energetic than (*n*_+_, *n*_−_) = (+3, +1) ones. It thus makes sense to apply the same arguments to configurations carrying more than one flux quantum. In the sector with negative *n*_−_, there are two possibilities to make a configuration that carries two flux quanta. Either to create two isolated (*n*_+_, *n*_−_) = (+1, −1) vortices carrying one flux quantum each or to create one (*n*_+_, *n*_−_) = (0, −2) two-quantum vortex. It turns out that a two-quantum vortex with smaller number of singularities is favoured as compared to two isolated single-quanta vortices. Figure 2(b) displays the ratio of the energies of two (isolated) *n*_−_ = −1 vortices and one *n*_−_ = −2 vortex. This ratio is always larger than one, thus implying that two-quanta vortices are energetically favoured as compared to two isolated single-quanta vortices. Note that the quantity displayed in Fig. 2(b), is actually also the ratio of first critical fields associated with single and double quanta vortices H_*c*1_(*n*_−_ = −1)/H_*c*l_(*n*_−_ = −2). Note also that smaller *H*_*c*1_ for a higher-flux vortex does not necessarily imply that such vortices will nucleate first in low magnetic field. That is, due to higher winding they carry larger magnetic flux and thus can have a higher potential barrier to enter the sample (compared with the discussion of Bean-Livingston barrier in single component superconductors^46^). The vortices (*n*_−_ = −1) and (*n*_−_ = −2) should interact differently with the Meissner currents and image charges, and thus even if the (*n*_−_ = −2) vortices have lower H_*c*l_, the interaction with the boundary may instead favour the entry of the vortices with (*n*_−_ = −1).

We also calculated the energy diagram similar to that in Fig. 2(b), but for vortices carrying three flux quanta *n*_−_ = −3 (data not shown). We found that unlike for *n*_−_ = −2, the *n*_−_ = −3 are not always stable. That is, in some regions of the parameter space the *n*_−_ = −3 is found, but in some other regions it decays into one single-quantum plus one double-quantum vortex. We find that for *n*_−_ < 0, the *n*_−_ = −2 skyrmions are all energetically favoured. This behaviour can already be anticipated from the last column in Fig. 1 where the four quanta *n*_−_ = −4 skyrmion seems to be a bound state of two *n*_−_ = −2 vortices. As the skyrmions with *n*_−_ < −2 are more energetic than those with *n*_−_ = −2, one can easily see that the *n*_−_ = −4 configuration can decay into two *n*_−_ = −2 vortices and thus reduce its total energy. We find that in the regions where the *n*_−_ = −3 vortices exist, they are more energetic than three isolated single quanta vortices (or one single plus one two-quantum vortex).

**Figure 3:**
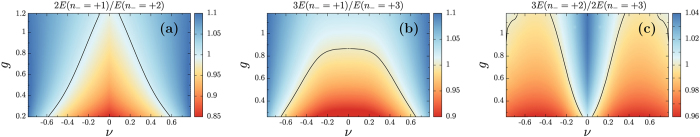
Ratio of the energies of multiple quanta vortices with *n*_−_ > 0 compared to isolated vortices carrying smaller number of flux quanta, as functions of the anisotropy parameter v and of the gauge coupling g. The solid line separates the regions where isolated vortices with smaller flux are preferred over single vortex carrying more flux quanta. Panel (**a**) and (**b**) respectively show the energetic behaviour of double (resp. triple) quantum vortex compared to isolated vortices with single flux quantum. Below the solid line, that is for more isotropic (small 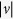) or more type-2 (small *g*), isolated single quanta vortices are energetically favoured. Panel (**c**) shows the energy ratio of two (isolated) triple-quanta vortices compared to three double quanta. This indicates subtle sublinear energy scaling with the number of flux quanta.

Although being energetically unfavoured, it is still instructive to consider the properties of vortices carrying multiple flux quanta, with *n*_−_ > 0. Diagrams in Fig. 3 show the ratio of the energies of multiple quanta vortices with *n*_−_ > 0, compared to that of isolated vortices carrying smaller flux. The situation for *n*_−_ > 0 is actually very different from that with *n*_−_ < 0. Panels (a) and (b) in Fig. 3 display the ratio of the energies of isolated single-quanta vortices with that of vortices carrying two and three flux quanta. Depending on the anisotropy parameter *v* and on the gauge coupling *g* this ratio can either be smaller or larger than one and the solid lines on the diagram show when these are degenerate in energy. Below the solid line, isolated single-quanta vortices are energetically favoured as compared to multi-quanta vortices. Above this line, these are the vortices carrying two or three flux quanta which are favoured. Thus tetragonal distortions of the Fermi surface (i.e. larger 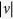) tend to favour *n*_−_ = +2 (and to a lesser extend *n*_−_ = +3), as compared to isolated *n*_−_ = +1 vortices. Note that the solid lines in panel (a) and (b) do not coincide. The panel (c) shows the comparison between three isolated double quanta and two isolated triple quanta vortices. Here again, depending on *v* and *g*, either can be preferred. This suggests complicated sublinear scaling of the energy with the number of flux quanta.

**Figure 4:**
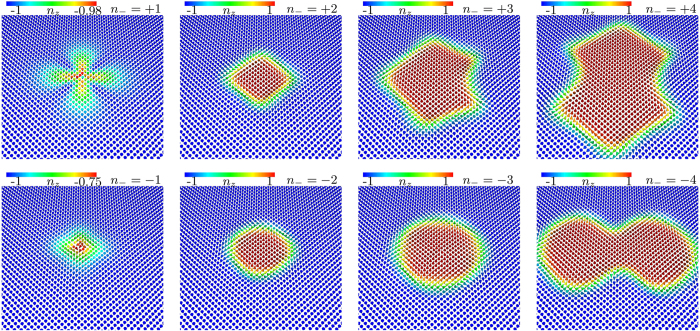
Pseudo-spin texture *n* defined as the projection of superconducting degrees of freedom onto spin-1/2 Pauli matrices. Different panels correspond to the solutions with different vorticity displayed in Fig. 1. First line shows vortices with *B*_*z*_ > 0 and the second line is for *B*_*z*_ < 0. The multi-quanta skyrmions are characterized by the topological charge (3) 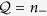. Single quanta vortices on the other hand, do not cover the whole sphere (i.e 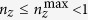) thus they have vanishing charge 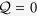.

The coreless nature of the two-quanta vortices implies that these have additional topological properties that are absent for single-quanta vortices. If the order parameter 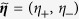 does not vanish 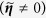, a pseudo-spin (unit) vector ***n*** can be defined as the projection of the order parameter on spin-1/2 Pauli matrices ***σ***: 
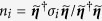
 (see detailed discussion of the pseudo-spin formalism for multi-component GL models in^47^). Figure 4 shows pseudo-spin texture for vortex solutions corresponding to those displayed in Fig. 1. Note that ***n*** is ill-defined for singular vortices, since there 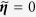 at the core (i.e. singularities in both components overlap). Coreless vortices on the other hand have well defined pseudo-spin projection which is a map 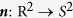. Since at spatial infinity, ***n*** = (0, 0, −1), the plane 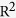 can be compactified to *S*^2^ so that the pseudo-spin becomes a map 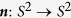. The homotopy invariants 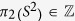 associated with such maps defines the integer-valued topological charge


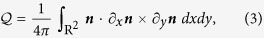


which can be used to classify various field configurations. Heuristically, 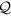 counts the number of times that the target sphere *S*^2^ is wrapped while covering the *xy*-plane. Singular configurations for which the pseudo-spin is not everywhere well-defined, have 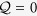. Non-singular solutions on the other hand, and in particular coreless vortices, have 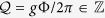 (where 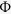 is the flux). For example the two-quanta vortices, which are coreless, are characterized by 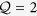. The fact that 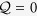 for singular vortices can easily be seen from the plot of the pseudo-spin texture Fig. 4. There ***n*** never reaches the north pole and thus do not fully cover the unit sphere.

## Discussion

Here, we reported a large-scale numerical investigation of the energy properties of isolated single and multiple quanta vortices/skyrmions in a Ginzburg-Landau model of chiral *p*-wave superconducting state. As pointed out previously, for a given ground-state chirality, vortices and anti-vortices are inequivalent. Thus we performed study for both orientations of the winding. The vortices with winding *n*_−_ = −1 in the dominant component are always preferred to those with winding *n*_−_ = +1. We also found that vortices carrying two flux quanta with *n*_−_ = −2 are always energetically favoured as compared to two isolated single-quanta vortices. Vortices with higher flux and negative *n*_−_, on the other hand, are either unstable or have higher energies per flux quantum. We also reported the structural and energetic properties of (meta-)stable skyrmions with various topological charge (i.e. for *n*_−_ > +1). The calculations show complicated sublinear scaling of the energy with the number of flux quanta that qualitatively agrees with previous works for a smaller parameter set in a related model^40^. Due to their very characteristic profiles of the magnetic field, their experimental observation, in e.g. scanning Hall and scanning SQUID experiments would provide a strong evidence of chiral *p*-wave superconductivity in the candidate materials described by the model (1). Note however that various aspects of microscopic physics may alter the form of the Ginzburg-Landau model (1), and in particular the balance between the different coefficients entering the free energy. This is currently a subject of ongoing studies, in connection with Sr_2_RuO_4_ (see e.g.^19^,^20^). Note added: after the completion of this work a study appeared reporting stable skyrmions as well as vortices, in this model, affected by mesoscopic effects in small samples^48^.

## Methods

In this work we used the dimensionless two-component Ginzburg-Landau theory Eq. (1) that was previously microscopically derived in the weak coupling limit (see for example refs 34,35). In this work, we focus on the properties of vortex solutions in the *xy*-plane and neglect the dependence over the third coordinate *z*. This means that our solutions are either purely two dimensional, or describe bulk configuration, assuming translation invariance along *z*-axis (and thus neglecting possible surface effects).

For the numerical investigation, the two-dimensional problem (1) is defined on a bounded domain 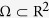. The boundary conditions for chiral *p*-wave superconductors can be very involved. Namely, in order to simulate chiral *p*-wave superconductors on a finite domain, a special attention has to be paid to boundary conditions to take into account edge currents properties. However, we are interested here in the intrinsic energetic properties of isolated defects. Thus we consider isolated vortices in large grids (such that there are no interactions with boundaries) and let the fields freely recover the ground-state. As a result, we probe the intrinsic structure and energy properties of vortices without any deformation originating from boundary behaviour. The simulation is run for a zero applied field (so that there are no Meissner currents), and the flux carrying solution is generated by a starting condition with a given winding of the dominant component. Because it enjoys topological protection, the (dominant) component cannot unwind by means of continuous transformations and thus topological properties (winding of the dominant component) are preserved by an energy minimization algorithm. Note that as simulations are run on a large but finite domain, there is still a possibility to change the topological sector, by moving the vortex across the boundary. This is possible, because without external fields there are no Meissner currents to prevent escape of a vortex. Note however that as we choose to work with large grids, the vortices in practice do not interact with boundaries, and thus they do not escape from the domain. The advantage of this choice is that it is guaranteed that obtained solutions are not affected by boundaries and that the calculated energies are those of isolated defects. The configurations displayed in the paper are close-up views of these defects.

For the actual numerical computation, the variational problem of minimizing the free energy is defined using a finite element formulation provided by the Freefem++ library^42^. Discretization within finite element formulation is done via a (homogeneous) triangulation over 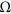, based on Delaunay-Voronoi algorithm. Functions are decomposed over a continuous piecewise quadratic basis on each triangle. The accuracy of such method is controlled through the number of triangles, (we typically used 3 ~ 6 × 10^4^), the order of expansion of the basis on each triangle (2nd order polynomial basis on each triangle), and also the order of the quadrature formula for the integral on the triangles. A nonlinear conjugate gradient algorithm is used to solve the variational nonlinear problem (i.e. to find the minima of 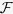). The algorithm is iterated until relative variation of the norm of the gradient of the functional 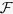 with respect to all degrees of freedom is less than 10^−8^ (we verified that for this value, the configuration does not evolve and the energy remains constant).

For the minimization procedure to lead to a configuration that has the expected topological properties, the starting field configuration should exhibit itself those desired topological properties. Although strictly speaking there is no infinite energy barrier between different topological sectors in finite domains, the barrier is finite but large enough to prevent any unwinding. Thus typically gradient minimization converges to the configuration that has the topological properties of the starting guess. In order to have efficient numerics, it is also important that the starting field configuration is the closest as possible to the minimal energy configuration. The initial field configuration carrying *N*_*v*_ flux quanta is prepared by using an ansatz which imposes phase winding of the dominant component (*η*_−_) around a given point (*x*_*k*_, *y*_*k*_):


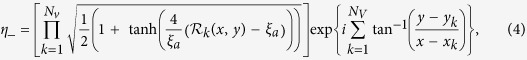



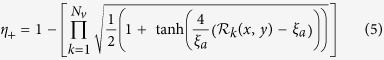


where 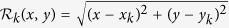 and *ξ*_*a*_ parametrizes the core size. The parametrization of *η*_+_, with nonzero density in the core enhances the convergence to form coreless defects. Finally, the starting configuration for the vector potential of the magnetic field ***A***, is determined by solving Ampère’s law equation **∇** × ***B*** + ***J*** = 0, for the supercurrent 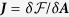 specified by the superconducting condensates given by (4). Being an equation linear in ***A***, this operation is rapidly solved. Once the starting configuration is constructed, all degrees of freedom are relaxed simultaneously.

## Additional Information

**How to cite this article**: Garaud, J. and Babaev, E. Properties of skyrmions and multi-quanta vortices in chiral *p*-wave superconductors. *Sci. Rep.* **5**, 17540; doi: 10.1038/srep17540 (2015).

## References

[b1] Volovik G. E. The Universe in a Helium Droplet, vol. 117 of International series of monographs on physics (Oxford University Press, 2009), 10.1093/acprof:oso/9780199564842.001.0001.

[b2] Mermin N. D. & Ho T.-L. Circulation and Angular Momentum in the A Phase of Superfluid Helium-3. Phys. Rev. Lett. 36, 594–597 (1976), 10.1103/PhysRevLett.36.594.

[b3] Anderson P. W. & Toulouse G. Phase Slippage without Vortex Cores: Vortex Textures in Superfluid ^3^He. Phys. Rev. Lett. 38, 508–511 (1977), 10.1103/PhysRevLett.38.508.

[b4] Mermin N. D. The topological theory of defects in ordered media. Rev. Mod. Phys. 51, 591–648 (1979), 10.1103/RevModPhys.51.591.

[b5] Thuneberg E. V. Identification of vortices in superfluid ^3^He-B. Phys. Rev. Lett. 56, 359–362 (1986), 10.1103/PhysRevLett.56.359. 10033167

[b6] Walmsley P. M., Cousins D. J. & Golov A. I. Critical Velocity of Continuous Vortex Nucleation in a Slab of Superfluid ^3^He-A. Phys. Rev. Lett. 91, 225301 (2003), 10.1103/PhysRevLett.91.225301. 14683246

[b7] WalmsleyP. M., WhiteI. J. & GolovA. I. Intrinsic Pinning of Vorticity by Domain Walls of 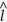 Texture in Superfluid ^3^He-A. Phys. Rev. Lett. 93, 195301 (2004), 10.1103/PhysRevLett.93.195301.15600844

[b8] Walmsley P. M. & Golov A. I. Chirality of Superfluid ^3^He-A. Phys. Rev. Lett. 109, 215301 (2012), 10.1103/PhysRevLett.109.215301. 23215597

[b9] Maeno Y. *et al.* Superconductivity in a layered perovskite without copper. Nature 372, 532–534 (1994).

[b10] Mackenzie A. P. & Maeno Y. The superconductivity of Sr2RuO4 and the physics of spin-triplet pairing. Rev. Mod. Phys. 75, 657–712 (2003), 10.1103/RevModPhys.75.657.

[b11] Joynt R. & Taillefer L. The superconducting phases of UPt_3_. Rev. Mod. Phys. 74, 235–294 (2002), 10.1103/RevModPhys.74.235.

[b12] Joynt R. Nature of the Lower Superconducting Transition in UPt_3_. EPL (Europhysics Letters) 16, 289 (1991), 10.1209/0295-5075/16/3/012.

[b13] Machida Y. *et al.* Twofold Spontaneous Symmetry Breaking in the Heavy-Fermion Superconductor UPt_3_. Phys. Rev. Lett. 108, 157002 (2012), 10.1103/PhysRevLett.108.157002. 22587277

[b14] Tsutsumi Y., Machida K., Ohmi T. & aki Ozaki M. A Spin Triplet Superconductor UPt_3_. Journal of the Physical Society of Japan 81, 074717 (2012), 10.1143/JPSJ.81.074717.

[b15] Volovik G. E. & Gorkov L. P. Superconducting classes in systems with heavy fermions. Zhurnal Eksperimentalnoi i Teoreticheskoi Fiziki 88, 1412–1428 (1985).

[b16] Sigrist M., Rice T. M. & Ueda K. Low-field magnetic response of complex superconductors. Phys. Rev. Lett. 63, 1727–1730 (1989), 10.1103/PhysRevLett.63.1727. 10040655

[b17] Matsumoto M. & Sigrist M. Quasiparticle States near the Surface and the Domain Wall in a p_x_ ± ip_y_-Wave Superconductor. Journal of the Physical Society of Japan 68, 994–1007 (1999), 10.1143/JPSJ.68.994.

[b18] Matsumoto M. & Sigrist M. Quasiparticle States near the Surface and the Domain Wall in a p_x_ ± ip_y_-Wave Superconductor. Journal of the Physical Society of Japan 68, 3120–3120 (1999), 10.1143/JPSJ.68.3120.

[b19] Bouhon A. & Sigrist M. Current inversion at the edges of a chiral p-wave superconductor. Phys. Rev. B 90, 220511 (2014), 10.1103/PhysRevB.90.220511.

[b20] Scaffidi T. & Simon S. H. Large Chern Number and Edge Currents in Sr_2_RuO_4_. Phys. Rev. Lett. 115, 087003 (2015), 10.1103/PhysRevLett.115.087003. 26340202

[b21] Vadimov V. & Silaev M. Predicted Nucleation of Domain Walls in p_x_ + ip_y_ Superconductors by a Z_2_ Symmetry-Breaking Transition in External Magnetic Fields. Phys. Rev. Lett. 111, 177001 (2013), 10.1103/PhysRevLett.111.177001. 24206513

[b22] Kirtley J. R. *et al.* Upper limit on spontaneous supercurrents in Sr_2_RuO_4_. Phys. Rev. B 76, 014526 (2007), 10.1103/PhysRevB.76.014526.

[b23] Hicks C. W. *et al.* Limits on superconductivity-related magnetization in Sr_2_RuO_4_ and PrOs_4_Sb_12_ from scanning SQUID microscopy. Phys. Rev. B 81, 214501 (2010), 10.1103/PhysRevB.81.214501.

[b24] Kallin C. & Berlinsky A. J. Is Sr_2_RuO_4_ a chiral p-wave superconductor? Journal of Physics: Condensed Matter 21, 164210 (2009), 10.1088/0953-8984/21/16/164210. 21825390

[b25] Knigavko A. & Rosenstein B. Magnetic Skyrmion Lattices in Heavy Fermion Superconductor UPt_3_. Phys. Rev. Lett. 82, 1261–1264 (1999), 10.1103/PhysRevLett.82.1261.

[b26] Knigavko A., Rosenstein B. & Chen Y. F. Magnetic skyrmions and their lattices in triplet superconductors. Phys. Rev. B 60, 550–558 (1999), 10.1103/PhysRevB.60.550.

[b27] Tokuyasu T. A., Hess D. W. & Sauls J. A. Vortex states in an unconventional superconductor and the mixed phases of UPt_3_. Phys. Rev. B 41, 8891–8903 (1990), 10.1103/PhysRevB.41.8891. 9993228

[b28] Sauls J. A theory for the superconducting phases of UPt_3_. Journal of Low Temperature Physics 95, 153–168 (1994), 10.1007/BF00754932.

[b29] Sauls J. A. & Eschrig M. Vortices in chiral, spin-triplet superconductors and superfluids. New Journal of Physics 11, 075008 (2009), 10.1088/1367-2630/11/7/075008.

[b30] Riseman T. M. *et al.* Observation of a square flux-line lattice in the unconventional superconductor Sr2RuO4. Nature 396, 242–245 (1998), 10.1038/24335.

[b31] Aegerter C. M. *et al.* Evidence for a square vortex lattice in Sr_2_RuO_4_ from muon-spin-rotation measurements. Journal of Physics: Condensed Matter 10, 7445 (1998), 10.1088/0953-8984/10/33/013.

[b32] Ray S. J. *et al.* Muon-spin rotation measurements of the vortex state in Sr_2_RuO_4_: Type-1.5 superconductivity, vortex clustering, and a crossover from a triangular to a square vortex lattice. Phys. Rev. B 89, 094504 (2014), 10.1103/PhysRevB.89.094504.

[b33] Curran P. J. *et al.* Vortex imaging and vortex lattice transitions in superconducting Sr_2_RuO_4_ single crystals. Phys. Rev. B 84, 104507 (2011), 10.1103/PhysRevB.84.104507.

[b34] Agterberg D. F. Vortex Lattice Structures of Sr_2_RuO_4_. Phys. Rev. Lett. 80, 5184–5187 (1998), 10.1103/PhysRevLett.80.5184.

[b35] Agterberg D. F. Square vortex lattices for two-component superconducting order parameters. Phys. Rev. B 58, 14484–14489 (1998), 10.1103/PhysRevB.58.14484.

[b36] Heeb R. & Agterberg D. F. Ginzburg-Landau theory for a p-wave Sr_2_RuO_4_ superconductor: Vortex core structure and extended London theory. Phys. Rev. B 59, 7076–7082 (1999), 10.1103/PhysRevB.59.7076.

[b37] Barash Y. S. & Mel’Nikov A. S. Possible existence of nonsingular-vortex in UPt_3_. ZhETF Pisma Redaktsiiu 51, 511 (1990).

[b38] Mel’nikov A. Phase transitions in vortex lattices of hexagonal exotic superconductors. Journal of Experimental and Theoretical Physics 74, 1059 (1992).

[b39] Ichioka M., Machida K. & Sauls J. A. Vortex States of Chiral p-wave Superconductors. *Journal of Physics: Conference Series* **400**, 022031 (2012), 10.1088/1742-6596/400/2/022031.

[b40] Garaud J. & Babaev E. Skyrmionic state and stable half-quantum vortices in chiral p-wave superconductors. Phys. Rev. B 86, 060514 (2012), 10.1103/PhysRevB.86.060514.

[b41] Sigrist M. & Ueda K. Phenomenological theory of unconventional superconductivity. Rev. Mod. Phys. 63, 239–311 (1991), 10.1103/RevModPhys.63.239.

[b42] Hecht F. New development in freefem++. J. Numer. Math. 20, 251–265 (2012), 10.1515/jnum-2012-0013.

[b43] Sauls J. A. Surface states, edge currents, and the angular momentum of chiral p-wave superfluids. Phys. Rev. B 84, 214509 (2011), 10.1103/PhysRevB.84.214509.

[b44] GaraudJ. & BabaevE. Vortex matter in 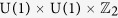 phase-separated superconducting condensates. Phys. Rev. B 90, 214524 (2014), 10.1103/PhysRevB.90.214524.

[b45] Ichioka M., Matsunaga Y. & Machida K. Magnetization process in a chiral p -wave superconductor with multidomains. Phys. Rev. B 71, 172510 (2005), 10.1103/PhysRevB.71.172510.

[b46] Bean C. P. & Livingston J. D. Surface Barrier in Type-II Superconductors. Phys. Rev. Lett. 12, 14–16 (1964), 10.1103/PhysRevLett.12.14.

[b47] Babaev E., Faddeev L. D. & Niemi A. J. Hidden symmetry and knot solitons in a charged two- condensate Bose system. Phys. Rev. B 65, 100512 (2002), 10.1103/PhysRevB.65.100512.

[b48] Fernández Becerra V., Sardella E., Peeters F. M. & Milošević M. V. Vortical versus skyrmionic states in mesoscopic p-wave superconductors. *ArXiv e-prints* (2015).

